# Complete Genome Sequence of an Alphabaculovirus from Choristoneura diversana

**DOI:** 10.1128/MRA.00051-20

**Published:** 2020-03-05

**Authors:** Jun Takatsuka

**Affiliations:** a Forestry and Forest Products Research Institute, Forest Research and Management Organization, Tsukuba, Ibaraki, Japan; Queens College

## Abstract

The genome sequence of a baculovirus isolated from Choristoneura diversana is 122,827 bp long and contains 150 putative open reading frames (ORFs). The virus is closely related to alphabaculoviruses isolated from insect species of the genus *Choristoneura*.

## ANNOUNCEMENT

Choristoneura diversana is a univoltine species belonging to the *Tortricidae* family, which is widely distributed in the Palearctic ecozone. Symptoms of nuclear polyhedrosis have been observed in larvae collected from *C. diversana* outbreak populations (1). A causal agent of nuclear polyhedrosis in *C. diversana* has not been reported; however, nucleopolyhedroviruses (NPVs) isolated from *Choristoneura* species such as C. muriana (ChmuNPV), C. occidentalis (ChocNPV), and C. rosaceana (ChroNPV) and two distinct NPVs from C. fumiferana (CfMNPV and CfDEFNPV) have been characterized (2–5). To characterize the nuclear polyhedrosis of *C. diversana*, an NPV was isolated from the insect, and the genome sequence was determined.

As per a previously described method (6), virions were purified from viral occlusion bodies prepared from three larval samples of *C. diversana* collected at a Todomatsu fir stand in Hokkaido, Japan, which had been preserved at the Forestry and Forest Products Research Institute since 1972. Viral genomic DNA was prepared as described previously (6). A paired-end sequence library with a 350-bp insert was prepared using a TruSeq DNA PCR-free kit (Illumina), and 101-base paired-end reads were generated on the HiSeq 2500 platform (Illumina) using a commercial sequencing service (Macrogen, Japan). Trimming of low-quality ends (Phred quality scores of <Q20) and adaptors was performed, and reads less than 40 bases long were filtered out using BBDuk2 (https://github.com/BioInfoTools/BBMap/blob/master/sh/bbduk2.sh) with the parameter settings ktrimright = t, k = 27, hdist = 1, qtrim = rl, trimq = 20, minlength = 40, trimoverlap = t, and minoverlap = 24. Assembly was conducted first using 5% of the total processed reads (1,052,696 of 21,053,934 reads) using *de novo* assembly in Geneious Prime 2019.2.3 (Biomatters) with default parameters of low sensitivity/fastest setting except that the “only use paired hits during assembly” option was used. This generated a contig with overlapping sequences at both ends, from which a circular contig was created. All processed reads were then used to assemble the circular contig, permitting 20,940,902 reads to be assembled and resulting in a mean coverage of 16,856× (standard deviation, 2,051×). An ambiguous region with lower coverage inside the *orf1629* gene was amplified using PCR, and the DNA was sequenced using Sanger sequencing. The PCR generated two amplicons (482 and 656 bp) containing repeat sequences. The genome sequence reported here used the sequence of the longer amplicon. Putative open reading frames (ORFs) encoding more than 50 amino acids were identified using the Glimmer 3 version 1.5 (7) algorithm trained for ChmuNPV ORF annotations using Geneious Prime 2019.2.3 (Biomatters) and were manually edited.

**FIG 1:**
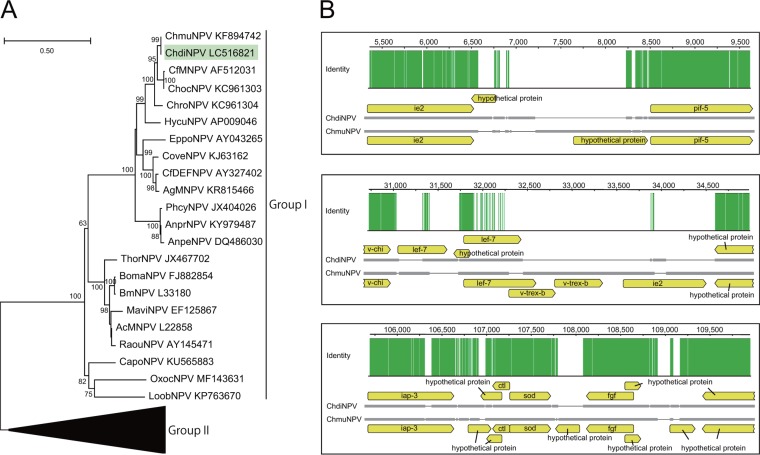
(A) Phylogenetic tree of alphabaculovirus. Amino acid sequences of *lef-8* and *pif-2* gene products were aligned with MUSCLE (11) using the default settings and concatenated after removing ambiguously aligned residues using the -gappyout command in trimAl version 1.2 (12). A maximum likelihood tree was inferred by employing the LG + G + I + F model of amino acid substitution (number of substitution rate categories, 4; gamma shape parameter, 0.796; proportion of invariable sites, 0.222) in the PhyML 3.0 Web server (13). The numbers at the nodes indicate Shimodaira-Hasegawa-like local support values (>50%). Branch termini are labeled according to virus names and GenBank accession numbers. ChdiNPV is shown on a green background. The group II alphabaculovirus cluster is compressed. (B) Divergent regions of the ChdiNPV and ChmuNPV genomes. In each panel, green bars in the identity graph show identical bases in the alignment columns between the viral genome sequences. A scale in the alignment, represented by the positions from A of the polyhedrin ATG start codon, is shown on the top of the identity graph. A schematic diagram of the alignment in each divergent region is shown by gray boxes and black lines below the identity graph. The gray boxes and black lines indicate nucleotide sequences and alignment gaps, respectively. Protein-coding sequences on the ChdiNPV and ChmuNPV genomes are indicated by yellow arrows above and below the alignment diagram, respectively.

The genome of the *C. diversana* NPV (ChdiNPV) was 122,827 bp long with a G+C content of 50.2% and was estimated to have 150 ORFs, including 6 ORFs unique to ChdiNPV. The remaining 144 ORFs had similarities to other baculovirus genes, including all baculovirus core genes. Global alignment using EMBOSS Stretcher (8) showed that the ChdiNPV genome had 95.4%, 81.6%, 81.2%, and 75.8% nucleotide identities to ChmuNPV, ChocNPV, CfMNPV, and ChroNPV, respectively. A blastp search with a cutoff E value of 1 × 10^−5^ using blast+ (9) found that ChdiNPV shared 140, 136, 136, and 136 ORFs with ChmuNPV, ChocNPV, CfMNPV, and ChroNPV, respectively. The mean amino acid identities of ChdiNPV putative proteins with orthologous proteins of the above-mentioned 4 viruses were 98.3%, 87.7%, 87.4%, and 84.4%, respectively. Phylogenic analysis using concatenated sequences of LEF-8 and PIF-2 indicates that ChdiNPV belongs to the group I alphabaculoviruses and is the closest to ChmuNPV in lineage, including ChocNPV and CfMNPV. ChroNPV was aligned to this lineage (Fig. 1A). Although the closest relationship identified was between ChdiNPV and ChmuNPV, several divergent regions were detected in an alignment conducted using progressiveMauve (10) implemented in Geneious Prime 2019.2.3 (Biomatters) using the default settings (Fig. 1B), which contributed to the discrepancy of the presence or absence of ORFs in each viral genome.

### Data availability.

The genome sequence of ChdiNPV has been deposited in DDBJ/EMBL/GenBank under the accession number LC516821, with raw sequence read data available at the DDBJ Sequence Read Archive under the accession number DRA009434.
